# Effects of Pitavastatin on Lipid Profiles in HIV-Infected Patients with Dyslipidemia and Receiving Atazanavir/Ritonavir: A Randomized, Double-Blind, Crossover Study

**DOI:** 10.1371/journal.pone.0157531

**Published:** 2016-06-15

**Authors:** Asita Wongprikorn, Chonlaphat Sukasem, Apichaya Puangpetch, Pawin Numthavej, Ammarin Thakkinstian, Sasisopin Kiertiburanakul

**Affiliations:** 1 Department of Medicine, Division of Infectious Diseases, Faculty of Medicine Ramathibodi Hospital, Mahidol University, Bangkok, Thailand; 2 Department of Pathology, Division of Pharmacogenomics and Personalized Medicine, Faculty of Medicine Ramathibodi Hospital, Mahidol University, Bangkok, Thailand; 3 Laboratory for Pharmacogenomics, Somdech Phra Debaratana Medical Center, Faculty of Medicine Ramathibodi Hospital, Mahidol University, Bangkok, Thailand; 4 Department of Clinical Epidemiology and Biostatistics, Faculty of Medicine Ramathibodi Hospital, Mahidol University, Bangkok, Thailand; Asociacion Civil Impacta Salud y Educacion, PERU

## Abstract

**Background:**

Dyslipidemia as a risk factor of cardiovascular disease is common especially in HIV-infected patients who are using protease inhibitors (PIs) including atazanavir. Pitavastatin has less drug-drug interactions and demonstrable efficacy in decreasing lipid levels in non HIV-infected individuals.

**Materials and Methods:**

This study was a randomized, double-blind, crossover study comparing the safety and efficacy of pitavastatin vs placebo in HIV-infected patients with dyslipidemia and receiving atazanavir/ritonavir (ATV/r). Patients were randomized to receive either placebo or pitavastatin for 12 weeks. The follow-up visits were every 4 weeks until the end of the study.

**Results:**

A total of 12 HIV-infected patients were enrolled to each study group. Of all, 14 (58%) patients were men and mean (standard deviation, SD) age was 48.1 (1.8) years. At 12 weeks of treatment with pitavastatin compared to placebo; mean [95% confidence interval (CI)] total cholesterol (TC) was 207 (187.3, 226.8) mg/dL vs 246.3 (226.5, 266) mg/dL (*p* <0.001); mean (95% CI) triglyceride (TG) was 351.3 (193.2, 509.4) mg/dL vs 279.1 (121, 437.2) mg/dL (*p* = 0.269); mean (95% CI) high density lipoprotein (HDL) was 45.3 (40.4, 50.2) mg/dL vs 44.2 (39.3, 49.1) mg/dL (*p* = 0.354); and mean (95% CI) low density lipoprotein (LDL) was 113.2 (100.4, 126) mg/dL vs 145.6 (132.8, 158.4) mg/dL (*p* <0.001). Mean liver enzyme and median creatine phosphokinase levels were not statistically significant between patients receiving placebo and pitavastatin.

**Conclusions:**

Pitavastatin decreases TC and LDL level at 12 weeks significantly and shows indifferent in hepatotoxicity and creatine phosphokinase levels compared to those of placebo. Thus, pitavastatin can be a good option of lipid-lowering agent in HIV-infected patients who are receiving ATV/r.

**Trial Registration:**

ClinicalTrials.gov NCT02442700

## Introduction

Dyslipidemia as a risk factor of cardiovascular disease (CVD) is common and attributed to HIV itself and/or the antiretroviral therapy (ART) used to treat HIV [1]. ART-related dyslipidemia is complex and involves various drug-induced effects, in association with hormonal and immunological influences superimposed upon genetic predisposition [2–3]. Protease inhibitors (PIs) are widely used as a combination therapy with other groups of antiretroviral drug due to their high genetic barrier property [4]. PI-associated dyslipidemia is also complex, multifactorial, and associated with multiple hepatocyte, adipocyte, and endothelial enzyme abnormalities [5]. Although atazanavir (ATV) has been shown to be associated with lesser dyslipidemia [6], ritonavir (RTV) which was frequently prescribed with PIs because of its booster effect in an increasing serum level of the PIs, can cause significantly increases in low density lipoprotein (LDL) and triglyceride (TG) level [5]. In Thailand, HIV drug resistance and the side effects of non-nucleoside reverse transcriptase inhibitors (NNRTIs) make PIs to be throughout used among patients with HIV infection.

Current guidelines support managing dyslipidemia in HIV-infected patients as in the general population [5, 7]. In patients with dyslipidemia, cardiovascular risk factors should be evaluated for lipid goals. If lipid goals are not achieved despite lifestyle modifications, the use of lipid lowering agents should be considered. A randomized, open-label clinical trial had proved significantly more effective in the management of ART-related dyslipidemia by adding lipid-lowering agents than the switching therapy from PIs to NNRTIs [8]. However, most of the lipid-lowering agents are metabolized by cytochrome P450 (CYP450), which are the same as most of the antiretroviral drugs. Thus, the drug-drug interactions have become a major concern in HIV-infected patients with dyslipidemia who need lipid-lowering agent treatment [9].

Pitavastatin, a recent HMG-CoA reductase inhibitor approved by FDA in 2009, has potent effect in decreasing total cholesterol (TC) and LDL level [10]. It is minimally metabolized by CYP450, but mainly undergoes glucuronidation which converted the substance to the inactive water-soluble form and subsequent elimination from the body through urine or feces. Therefore, the incidence of any drug interactions is reduced compared with other lipid-lowering agents used in HIV-infected patients [11]. The INTREPID trial is the only study of pitavastatin that demonstrated a superior reduction in LDL with safety profile to use in HIV-infected patients in the United States [12]. However, the INTREPID trial did not emphasize the issue of drug-drug interaction between PIs and HMG-CoA reductase inhibitor. The aims of study are to determine the efficacy and safety of pitavastatin in HIV-infected Thai patients with dyslipidemia who are receiving ATV/RTV (ATV/r).

## Materials and Methods

### Study design, setting, and patients

A randomized, double-blind, crossover study was conducted in outpatient clinic at Ramathibodi Hospital, a 1,200-bed university hospital in Thailand. The study period was from March 2014 to January 2015. Written consent was obtained from all participants. The protocol was approved by the local institutional review board (IRB) of Faculty of Medicine Ramathibodi Hospital, Mahidol University.

Inclusion criteria consists of patients aged ≥18 years, had confirmed HIV infection, on ART including 2 nucleoside reverse transcriptase inhibitors (NRTIs) or 1 NRTI and 1 NNRTI plus ATV 300 mg and RTV 100 mg each day in the regimens that were not changed within 12 weeks before the randomization, had TC level between 200 and 500 mg/dL and/or LDL between 130 and 400 mg/dL, and no lipid-lowering agent or discontinued the lipid-lowering agent for at least 4 weeks prior to randomization. Exclusion criteria consists of patients with the history of pitavastatin and the constituent of the drugs allergy, known history of myocardial infarction and/or ischemic stroke within 4 weeks prior to the randomization, abnormal aspartate aminotransferase (AST) and alanine aminotransferase (ALT) with level ≥5 times if asymptomatic or ≥3 times of upper normal limit (UNL) if symptomatic, pregnancy or breastfeeding, and currently on cyclosporine or other drugs which had major drug interactions with pitavastatin.

### Interventions

Any patients who visited the outpatient clinic and fulfilled the inclusion criteria were invited to participate to the study until the number of the patients were completed of 24. Then, they were randomly assigned either to pitavastatin or placebo. The sequence was generated using an internet-based randomization generator with a block size of 2 and concealed the sequence in the closed envelopes by a statistician until interventions were assigned. All patients were served as his or her own control, so the patients in each group received the study drug for 12 weeks from the physician, and then discontinue the study drug for 2 weeks (washout period) before switching to another treatment arm for 12 weeks. The placebo which was produced from the same manufacture of pitavastatin was identical to pitavastatin. Both drug and placebo were prepared before the initiation of the study and packed into identical containers by a pharmacist. Both physician and participants were blinded. Patients were randomly assigned to receive a sequentially numbered container that contained 28 tablets of pitavastatin (2 mg) or placebo with the instruction to take 1 tablet each day until the appointment visit.

On the first day of the enrollment, baseline characteristics and initial laboratory evaluation including TC, TG, LDL, high density lipoprotein (HDL), fasting blood sugar (FBS), AST, ALT, and creatinine (Cr) were collected. Assessments of changes of lipid profiles (TC, TG, LDL, and HDL), as well as clinical and laboratory safety evaluations including AST and ALT were performed every 4-week-visit. Creatine phosphokinase (CPK) and ATV level were evaluated additionally at 12 weeks and the end of study. The ATV level was performed by using high performance liquid chromatography (HPLC) assay [13]. Pill counts at each patient’s clinic visit was used for determination of compliance to the study drug.

This study was registered in ClinicalTrials.gov under registration number NCT02442700, but registered after recruitment of participants due to unplanned registry. The authors confirm that all ongoing and related trials for this drug/intervention are registered.

### Study outcomes

The primary outcome was efficacy of pitavastatin in HIV-infected patients with dyslipidemia and receiving ATV/r. Efficacy was measured by level of TC, TG, LDL, and HDL that decreased after pitavastatin treatment. Pitavastatin was considered efficient when it could decrease TC, TG, LDL, or HDL significantly compared to placebo.

The secondary outcome was safety of pitavastatin in HIV-infected patients. Safety assessments included clinical and laboratory evaluations, including AST, ALT, and CPK level. Safety clinical was defined by Food and Drug Administration (FDA); grade 1 mild symptoms; grade 2 moderate symptoms with limiting age-appropriate instrumental activities of daily living (ADL); grade 3 severe symptoms with limiting self- care ADL, but not immediately life-threatening; grade 4 life-threatening consequences; and grade 5 death related to adverse event. Pitavastatin was determined safe if AST, ALT and/or CPK level was not increased significantly compared to placebo.

### Statistical methods

We used the PS program version 3.0.43 with paired design t-test for calculating the sample size. From the previous study [14], we use the difference of means and standard deviation (SD) of lipid profiles of 20. Assuming drop-out rate of 20%, statistical power of 80% and type I error less than 5%, a total of 24 evaluable patients were needed to be enrolled.

Statistical analysis was performed with Stata 13.1 (StataCorp. 2013. *Stata Statistical Software*: *Release 13*. College Station, TX: StataCorp LP.). Multilevel mixed effect linear regression model was used in analyzing the efficacy and safety of pitavastatin on lipid profiles compared to placebo. We used mean (95% confidence interval, CI), mean difference (95% CI), and median (IQR) to report the results. All p-values were two-tailed with those less than 0.05 were considered statistically significant.

## Results

### Patients

**Fig 1:**
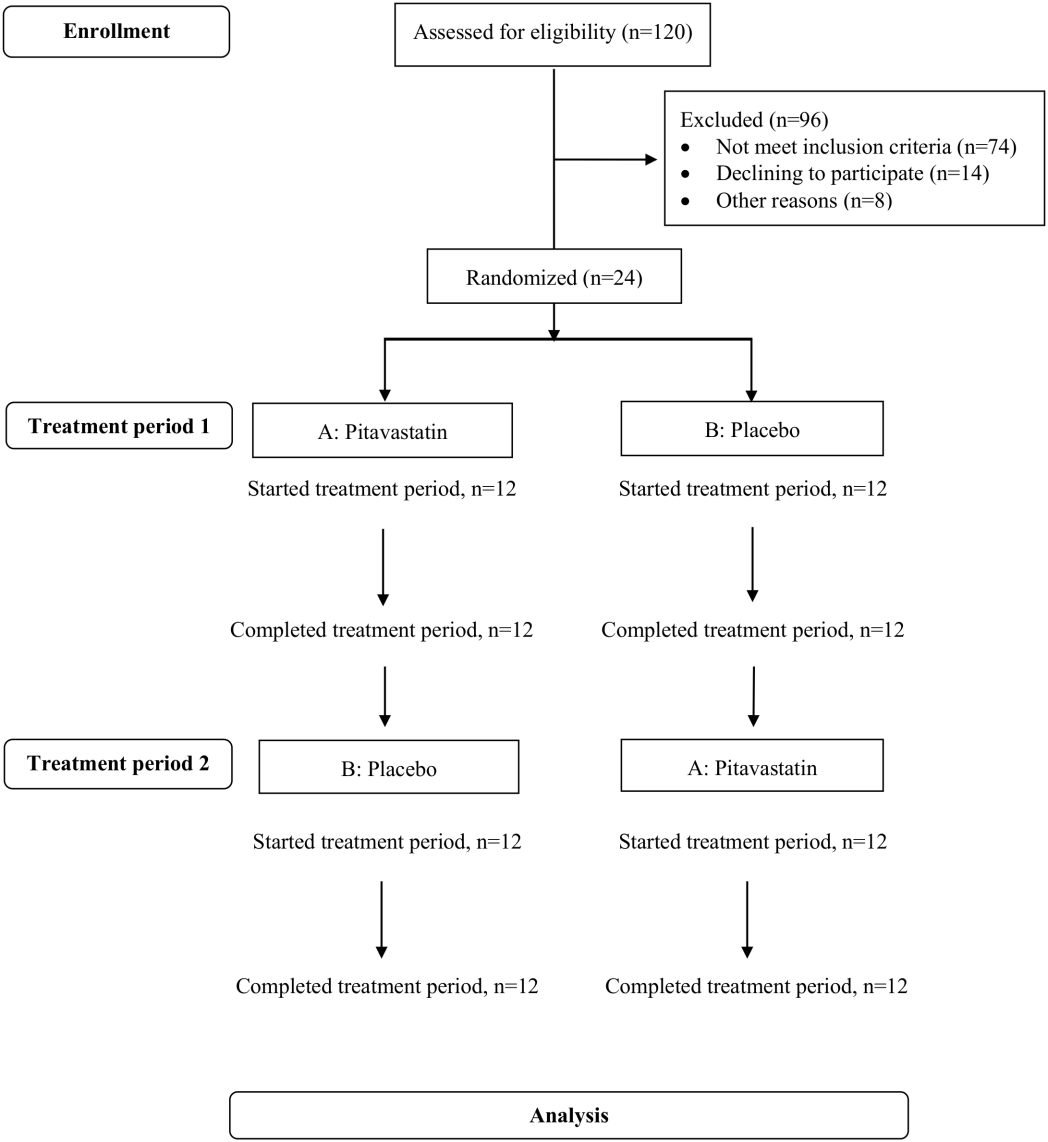
Flowchart of Subjects Participating in the Different Phases of Randomized Crossover Trial.

One hundred and twenty patients were assessed for eligibility, 96 patients were excluded due to not meet inclusion criteria and declining to participate the study “Fig 1”. Of 24 patients enrolled, all was followed up through the study period. Mean (standard deviation, SD) age was 48.1 (1.8) years and 14 (58.3%) patients were men. No difference of baseline characteristics between 12 patients who received placebo first and 12 patients who received pitavastatin first “Table 1”.

**Table 1:** Baseline Characteristics, Patients Who Received Placebo First Compared to Those That Received Pitavastatin First.

Characteristics	Placebo(n = 12)	Pitavastatin(n = 12)
Age, years*	46.7 (6.8)	49.6 (10.6)
Sex, n (%)		
Male	6 (50)	8 (66.7)
Female	6 (50)	4 (33.3)
Body mass index, kg/m^2^*	22.5 (3.4)	23.8 (2.7)
Underlying conditions, n (%)		
No	8 (66.7)	4 (33.3)
Dyslipidemia	2 (16.7)	4 (33.3)
Chronic hepatitis B and C virus infection	2 (16.7)	2 (16.7)
Others	0 (0)	2 (16.7)
Cardiovascular risk factors, n (%)^$^		
<2	11 (91.7)	7 (58.3)
≥2	1 (8.3)	5 (41.7)
Baseline creatinine, mg/dL*	0.9 (0.2)	0.9 (0.2)
Baseline FBS, mg/dL*	97.1 (10.5)	100.8 (10.1)
Baseline CD4 cell counts, cells/mm^3^*	718.1 (181.2)	641.9 (196.5)
HIV RNA <40 copies/mL, n (%)	11 (91.7)	12 (100)
Duration of ATV/r use, months^#^	36 (24–36)	42 (30–54)
ARV regimens combined with ATV/r, n (%)		
TDF + FTC/3TC	6 (50)	5 (41.7)
TDF + other NRTIs (exclude 3TC/FTC)	2 (16.7)	2 (16.7)
No TDF in backbone	4 (33.3)	5 (41.7)

*Mean (SD),

^$^current smoking, systolic blood pressure ≥140 mmHg or on antihypertensive drugs, HDL <40 mg/dL, first-degree relative <55 years in male and <65 years in female, and age >45 years in male or >55 years in female,

^#^median (IQR)

3TC: lamivudine, ARV: antiretroviral, ATV/r: atazanavir/ritonavir, FBS: fasting blood sugar, FTC: emtricitabine, IQR: interquartile range, SD: standard deviation, TDF: tenofovir disoproxil fumarate.

### Efficacy of pitavastatin

At 12 weeks of treatment with pitavastatin compared to placebo; mean (95% CI) TC was 207 (187.3, 226.8) mg/dL vs 246.3 (226.5, 266) mg/dL (*p* <0.001); mean (95% CI) TG was 351.3 (193.2, 509.4) mg/dL vs 279.1 (121, 437.2) mg/dL (*p* = 0.269); mean (95% CI) HDL was 45.3 (40.4, 50.2) mg/dL vs 44.2 (39.3, 49.1) mg/dL (*p* = 0.354); and mean (95% CI) LDL was 113.2 (100.4, 126) mg/dL vs 145.6 (132.8, 158.4) mg/dL (*p* <0.001) “Table 2”. During the use of 2 mg of pitavastatin, 18 patients could reach the target LDL less than 130 mg/dL.

**Table 2:** Lipid Measurements, Patients Who Received Placebo First Compared to Those That Received Pitavastatin First.

Period of treatment	Lipid variables* (mg/dL)	Placebo (n = 12)	Pitavastatin (n = 12)	Mean difference (95% CI)	P-value
**Baseline**	TC	257.6 (237.9, 277.3)	239.9 (220.2, 259.6)	17.8 (2.6, 32.9)	0.022
	TG	350 (191.9, 508)	282 (123.9, 440)	68 (60.2, 196.1)	0.299
	LDL	146.3 (133.6, 159.1)	144.7 (131.9, 157.4)	1.7 (-9.2, 12.6)	0.764
	HDL	44.8 (39.9, 49.7)	43 (38.1, 47.9)	1.8 (0.6, 4.2)	0.140
**4 weeks**	TC	246.6 (226.9, 266.3)	201.3 (181.5, 221)	45.4 (30.2, 60.6)	<0.001
	TG	292.5 (134.4, 450.5)	246.5 (88.4, 404.5)	46 (-82.2, 174.2)	0.482
	LDL	142.5 (129.7, 155.3)	111.6 (98.8, 124.4)	30.9 (20, 41.8)	<0.001
	HDL	43.5 (38.7, 48.4)	43.5 (38.6, 48.3)	0.1 (-2.3, 2.5)	0.945
**8 weeks**	TC	255.2 (235.4, 274.9)	202.3 (182.6, 222.1)	52.8 (37.6, 68)	<0.001
	TG	334 (176, 492.1)	250.8 (92.7, 408.8)	83.3 (44.9, 211.5)	0.203
	LDL	145.1 (132.3, 157.9)	111.5 (98.7, 124.3)	33.6 (22.7, 44.5)	<0.001
	HDL	43.7 (38.8, 48.6)	44.9 (40, 49.8)	-1.2 (-1.2, 3.5)	0.337
**12 weeks**	TC	246.3 (226.5, 266)	207 (187.3, 226.8)	39.2 (24, 54.4)	<0.001
	TG	279.1 (121, 437.2)	351.3 (193.2, 509.4)	-72.2 (-200.4, 56)	0.269
	LDL	145.6 (132.8, 158.4)	113.2 (100.4, 126)	32.4 (21.5, 43.3)	<0.001
	HDL	44.2 (39.3, 49.1)	45.3 (40.4, 50.2)	1.1 (-1.3, 3.5)	0.354

*Mean (95% CI).

### Safety of pitavastatin

Most of the patients (20 of 24, 83.3%) had 100% compliance. Three (12.5%) patients and 1 (4.2%) patient had compliance of 89.3% and 82.1%, respectively. No patient who received pitavastatin reported any adverse event including drug rash, myalgia, joint pain, or diarrhea. Compared to placebo, after 12 weeks of treatment with pitavastatin; mean (95% CI) AST was 40.8 (32.3, 49.2) U/L vs 39.5 (31.1, 48.0) U/L (p = 0.695); mean (95% CI) ALT was 72.5 (54.6, 90.3) U/L vs 64.2 (46.4, 82.1) U/L (p = 0.147); median (IQR) CPK level was 119 (41–516) U/L vs 103 (34–472) U/L (p = 0.555); and median (IQR) ATV level was 1.07 (0.58–1.535) mg/L vs 0.875 (0.445–1.41) mg/L (p = 0.689) (normal range 0.15–0.85 mg/L).

## Discussion

In this a randomized, double blind, crossover study, we found that pitavastatin has efficacy on significant reduction of TC and LDL after 12 weeks of treatment without clinical and laboratory adverse event compared to placebo in HIV-infected Thai patients with dyslipidemia and receiving ATV/r.

This study confirmed the previous trials in both normal population [15] and HIV-infected patients [12] demonstrating that pitavastatin is effective in lowering LDL and TC level. In the INTREPID trial, the baseline characteristics of HIV-infected patients are similar to those of our patients including age, CD4 cell counts and baseline lipid profiles, except the lower proportion of male patients and the lower body mass index (BMI) in our patients. Pitavastatin has a novel cyclopropyl group on its base structure, which is more effective inhibition of HMG-CoA reductase enzyme to inhibit cholesterol production and potentially affords greater LDL clearance and reduction of TC [10], leading to the reduction of both TC and LDL levels even though the lower dosage of pitavastatin used in our study compared with those used in the INTREPID trial. Although at baseline, TC level was lower in group starting with pitavastatin and indifferent LDL level between 2 groups, we demonstrated the effects of pitavastatin on both TC and LDL reduction since 4 weeks after treatment and the effect maintain through 12 weeks of treatment.

The majority of side effects of statin therapy were liver transaminase elevations and myopathy which were comparable among the various statins including pitavastatin [16]. Liver transaminase elevations were reported within the first 12 weeks of statin therapy at incidences of up to 1% which were dose related [17–18]. Statin-associated myopathy, usually presents within a few weeks to more than 2 years after statin therapy [19]. No adverse event was observed in the present study compared to the INTREPID trial. In the INTREPID trial, the incidence of drug-related emergent adverse events was 11.1% and 4.8% have to discontinue pitavastatin during the study, 3.2% have ALT level more than 2 times of UNL and 1.6% have CPK level more than 5 times of UNL. This might be due to the lower dosage of pitavastatin which we used (2 mg vs 4 mg). Four (16.7%) of our patients had chronic hepatitis B virus (HBV) and hepatitis C virus (HCV) co-infection, however there was no hepatotoxicity among these patients.

In healthy volunteer, ATV increases pitavastatin level (area under curve increased 31%, and C_max_ increased 60%) by unknown mechanism, but no significant change in ATV level by pitavastatin [17]. C_min_ was used to monitor safety and correlation in HIV-infected patients in some studies [20–21]. In our study, pharmacokinetics (PK) of atazanavir was not changed following pitavastatin and placebo administration. C_min_ value of ATV with and without pitavastatin in our study was within normal range (0.15–0.85 mg/L) which can imply that pitavastatin has no effect to ATV level in HIV-infected patients.

The strength of this study is a first double-blind, randomized controlled, crossover study to demonstrate efficacy and safety of pitavastatin in HIV-infected patients. The influence of confounding covariates is reduced because each crossover patient serves as his or her own control. There was no drop out of patients before the end of treatment at week 12. This is the first study to report ATV levels in HIV-infected patients who were receiving pitavastatin. Limitation of the study is we did not adjust pitavastatin dosage according to lipid profiles, thus mean value of TC could not be lower than 200 mg/dL. Furthermore, pitavastatin was compared to placebo because the aim of this study is to demonstrate efficacy and safety of the drug, which safety is the great concern owing to the drug-drug interaction issue.

## Conclusions

In conclusions, pitavastatin demonstrates the efficacy for TC and LDL reduction since 4 weeks and the effect maintain through 12 weeks of treatment. Pitavastatin shows neither hepatotoxicity nor increasing CPK level, thus pitavastatin can be an option of lipid-lowering agent in HIV-infected patients who are receiving ATV/r.

## Supporting Information

S1 Fig Full Trial Protocol for Ethical Consideration by the Local Institutional Review Board (IRB) of Faculty of Medicine Ramathibodi Hospital, Mahidol University. (DOCX)

S2 Fig Documentary Proof of Ethical Clearance Committee on Human Rights Related to Research Involving Human Subjects Faculty of Medicine Ramathibodi Hospital, Mahidol University. (DOCX)

S3 Fig CONSORT 2010 Checklist of Information to Include When Reporting a Randomized Trial. (DOC)

S4 Fig Full Trial Protocol for Ethical Consideration by the Local Institutional Review Board (IRB) of Faculty of Medicine Ramathibodi Hospital, Mahidol University in Thai Language. (DOC)

S5 Fig Case Record Form. (DOCX)
