# Supplementary material for: Effects of Pitavastatin on Lipid Profiles in HIV-Infected Patients with Dyslipidemia and Receiving Atazanavir/Ritonavir: A Randomized, Double-Blind, Crossover Study
Source: PLoS One. 2016 Jun 15;11(6):e0157531. doi: 10.1371/journal.pone.0157531 (PMC4909195; doi:10.1371/journal.pone.0157531)
Supplement: S1 Fig — (DOCX) [file pone.0157531.s001.docx]

**S1 Fig. Full Trial Protocol for Ethical Consideration by the Local Institutional Review Board (IRB) of** **Faculty of Medicine, Ramathibodi Hospital, Mahidol University**

1. **Title:** Effects of Pitavastatin on Lipid Profiles in HIV-infected Patients with Dyslipidemia and Receiving Atazanavir/Ritonavir: A Randomized, Double-blind, Crossover Study
2. **Author**

Asita Wongprikorn, MD

Qualification: Faculty of Medicine, Siriraj Hospital, Mahidol University

Contact address: Faculty of Medicine, Ramathibodi Hospital, Mahidol University

Tel. 09-1774-5573, 08-7930-7667, 46147

1. **Advisor**

Assoc. Prof. Sasisopin Kiertiburanakul, MD, MHS

Qualification: Faculty of Medicine, Ramathibodi Hospital, Mahidol University; Master of Health Science, Johns Hopkins University

Contact address: Faculty of Medicine, Ramathibodi Hospital, Mahidol University

Tel. 0-2201-0033, 08-1808-2223

1. **Rationale**

Cardiovascular disease is common cause of death in HIV-infected patients, mortality rate of cardiovascular disease in HIV-infected patients in Europe is increasing from 8% in 2000 to 10% and 14% respectively in 2005 and 2010 [1]. Dyslipidemia as a risk factor of cardiovascular disease (CVD) is common and attributed to HIV itself and/or the antiretroviral therapy (ART) used to treat HIV. ART-related dyslipidemia is complex and involves various drug-induced effects, in association with hormonal and immunological influences superimposed upon genetic predisposition [2]. Protease inhibitors (PIs) are widely used as a combination therapy with other groups of antiretroviral drug due to their high genetic barrier property compared with nucleoside reverse transcriptase inhibitors (NRTIs) and non-nucleoside reverse transcriptase inhibitors (NNRTIs) [3]. In Thailand, HIV drug resistance and the side effects of NNRTIs make PIs to be throughout used among patients with HIV infection. PIs treatment can result in dyslipidemia in a significant proportion of patients [2]. Although atazanavir (ATV) has been shown to be associated with lesser dyslipidemia [4], ritonavir (RTV) which was frequently prescribed with PIs because of its booster effect in an increasing serum level of the PIs, can cause significantly increases in low density lipoprotein (LDL) and triglyceride (TG) level [5].

A randomized, open-label clinical trial had proved significantly more effective in the management of ART-related dyslipidemia by adding lipid-lowering agents than the switching therapy from PIs to NNRTIs [6]. However, most of the lipid-lowering agents are metabolized by cytochrome P450 (CYP450), which are the same as most of the antiretroviral drugs [7]. Thus, the drug-drug interactions have become a major concern in HIV-infected patients with dyslipidemia who need lipid-lowering agent treatment.

Pitavastatin, a new HMG-CoA reductase inhibitor approved by FDA in 2009, has potent effect in decreasing total cholesterol (TC) and LDL level. It is minimally metabolized by CYP450, but mainly undergoes glucuronidation which converted the substance to the inactive water-soluble form and subsequent elimination from the body through urine or feces [8]. Therefore, the incidence of any drug interactions is reduced compared with other lipid-lowering agents used in HIV-infected patients. The INTREPID trial is the only study of pitavastatin that demonstrated a superior reduction in LDL with safety profile to use in HIV-infected patients in the United States compared with pravastatin [9]. However, most of HIV-infected Thai patients lack of the assessable to lipid-lowering agents besides simvastatin because of the high cost of original drugs. The aims of study are to determine the efficacy and safety of pitavastatin in HIV-infected Thai patients with dyslipidemia who are receiving ATV/RTV (ATV/r).

1. **Objectives**
   1. Primary objective: efficacy of pitavastatin in HIV-infected patients with dyslipidemia and receiving ATV/r
   2. Secondary objective: safety of pitavastatin in HIV-infected patients
2. **Materials and Methods**

A randomized, double-blind, crossover study is conducted in outpatient clinic at Ramathibodi Hospital, a 1,200-bed university hospital in Thailand. The study period is from March to December 2014.

*Methods*

1. HIV-infected patients in outpatient clinic at Ramathibodi Hospital which received ATV/r are evaluated by the research provider.
2. If HIV-infected patients are compatible with inclusion criteria, the research provider will provide information about the research for participants.
3. Baseline characteristics [sex, age, body mass index (BMI), underlying conditions, cardiovascular risk factor, lastest CD4 cell count and HIV viral load, antiretroviral regimen combined with ATV/r and duration of ATV/r use] are collected from participants.
4. Baseline fasting blood sugar (FBS), aspartate aminotransferase (AST), alanine aminotransferase (ALT), Creatinine (Cr) are collected from each participant.
5. Participants are allocated to receive placebo or pitavastatin by computer-generated randomization by block of 2 under sealed envelope. Both research provider and participants are blinded to receive the study drug.
6. Follow-up visit is every 4 weeks for history, physical examination, and blood collection.
7. Analysis of the results to evaluate efficacy and safety of pitavastatin is compared with placebo

*Medical data collection*

The research provider will collect the participant data in case report form (CRF)

**7. Protocol flow chart**

24 HIV-infected patients with dyslipidemia and receiving atazanavir/ritonavir

Baseline lipid profile [total cholesterol (TC), triglyceride (TG), LDL, and HDL], fasting blood sugar, AST, ALT and serum creatinine are collected before randomization.

2 weeks

Stop pitavastatin

Stop placebo

Stop pitavastatin and placebo in both groups of patients

*4 weeks follow-up visit

12 weeks

Placebo is administered.

Pitavastatin is administered.

12 weeks

**Group A**

Pitavastatin is administered.

**Group B**

Placebo is administered.

*every 4 weeks follow-up visit

Double-blind randomization with a block size of 2

*Pills count, history and physical examination are evaluated for side effects of the study drugs and lipid

profile (TC, TG, LDL, HDL), AST, ALT are collected every visit.

Atazanavir level and creatine kinase are collected at 12 and 24 weeks of randomization.

1. **Number of participants inclusion and exclusion criteria**

Total of 24 participants was included. (sample size calculation in the appendix)

8.1 Inclusion criteria

- Delight to participate in the study
- Has confirmed HIV infection
- Age ≥18 years
- On ART including 2 nucleoside reverse transcriptase inhibitors (NRTIs) or 1 NRTI and 1 NNRTI plus ATV 300 mg and RTV 100 mg each day in the regimens that are not changed within 12 weeks before the randomization
- Has TC level between 200 and 500 mg/dL and/or LDL between 130 and 400 mg/dL
- No any lipid-lowering agent or discontinue the lipid-lowering agent for at least 4 weeks prior to randomization

8.2 Exclusion criteria

- Patients with the history of pitavastatin and the constituent of the drugs allergy
- Known history of myocardial infarction and/or ischemic stroke within 4 weeks prior to the randomization
- Abnormal AST and ALT with level ≥5 times if asymptomatic or ≥3 times of upper normal limit (UNL) if symptomatic
- Pregnancy or breastfeeding
- Currently on cyclosporine or other drugs which has major drug interactions with pitavastatin
- Not willing to participate in the study

1. **Period of study:** Total 10 months since March to December 2014
2. **Estimated Risk for Participants**

• Blood collection is needed for total 8 times with 10-15 mL each time, then painful symptom, bruise or nearly syncope can be happened in each participant.

1. **Estimated Benefit from the Study**

• This study may show the efficacy and safety of pitavastatin as a good option to use in HIV-infected patients with dyslipidemia and receiving ATV/r

1. **Ethical Consideration**

- This study is proceeded following the Declaration of Helsinki.
- Participation is under willing of participants.
- Participants can refuse to participate this study without disadvantage in HIV treatment.
- The research provider will conceal the information of all participants.

1. **Compensation for participants**

Participants will receive the travelling expense for 300 Baht each time and cost of blood collection every visit.

1. **Research grant**

Grant support is received from Faculty of Medicine Ramathibodi Hospital.

Pitavastatin and placebo are supported by Kowa Company Limited (Japan)**.**

1. **Patient/participant information sheet:** Document 1
2. **Inform consent:** Document 2

Asita Wongprikorn, MD

Sasisopin Kiertiburanakul, MD, MHS

**References**

Henard S, Roussillon C, Bonnet F, et al. Cardiovascular-related deaths in HIV positive patients between 2000 and 2010: Agence Nationale de Recherche sur le Sida EN20 Mortalite 2010 Survey. 20^th^ Conference on Retroviruses and Opportunistic Infections. March 3-6, 2013; Atlanta. Abstract 1048.

Estrada V, Portilla J. Dyslipidemia related to antiretroviral therapy. AIDS Rev 2011;13:49-56.

Arora D, Dixit NM. Timing the emergence of resistance to anti-HIV drugs with large genetic barriers. PLoS Comput Biol 2009;5(3): e1000305.

Luwdgren JD, Battegay M, Behrens G, Wit SD, Guaraldi G, Katlama C, et al. European AIDS Clinical Society (EACS) guidelines on the prevention and management of metabolic diseases in HIV. HIV Med 2008;9:72-81.

Dube PM, Stein JH, Aberg JA, Fichtenbaum CJ, Gerber JG, Tashima KT, et al. Guidelines for the evaluation and management of dyslipidemia in Human Immunodeficiency Virus (HIV)-infected adults receiving antiretroviral therapy: Recommendations of the HIV Medicine Association of the Infectious Disease Society of America and the Adult AIDS Clinical Trials Group. Clin Infect Dis 2003;37:613-27.

Calza L, Manfredi R, Colangeli V, Tampellini L, Sebastiani T, Pocaterra D, et al. Substitution of nevirapine or efavirenz for protease inhibitor versus lipid-lowering therapy for the management of dyslipidemia. AIDS 2005;19:1051-8.

Neuvonen DJ, Niemi M, Backman JT. Drug interactions with lipid-lowering drugs mechanisms and clinical relevance. Clin Pharmacol Ther 2006;80:565-81.

Full prescribing information of pitavastatin, reference ID: 3090715.

Sponsellar CA, Morgan RE, Campbell SE, Kryzhanovski VA, Kartman CE, Aberg JA, et al. Pitavastatin 4 mg provides superior LDL-C reduction versus pravastatin 40 mg over 12 weeks in HIV-infected adults with dyslipidemia, the INTREPID trial. 20^th^ Conference on Retroviruses and Opportunistic Infections. March 3, 2013; Atlanta. Abstract 139.

**Appendix**

*Sample size calculation*

We used the PS program version 3.0.43 with paired design t-test for calculating the sample size. From the previous study [14], we use the difference of means and standard deviation (SD) of lipid profiles of 20. Assuming drop-out rate of 20%, statistical power of 80% and type I error less than 5%, a total of 24 evaluable patients were needed to be enrolled.
