# Supplementary material for: Effects of Pitavastatin on Lipid Profiles in HIV-Infected Patients with Dyslipidemia and Receiving Atazanavir/Ritonavir: A Randomized, Double-Blind, Crossover Study
Source: PLoS One. 2016 Jun 15;11(6):e0157531. doi: 10.1371/journal.pone.0157531 (PMC4909195; doi:10.1371/journal.pone.0157531)

**S2 Fig. Documentary Proof of Ethical Clearance Committee on Human Rights Related to Research Involving Human Subjects Faculty of Medicine Ramathibodi Hospital, Mahidol University**


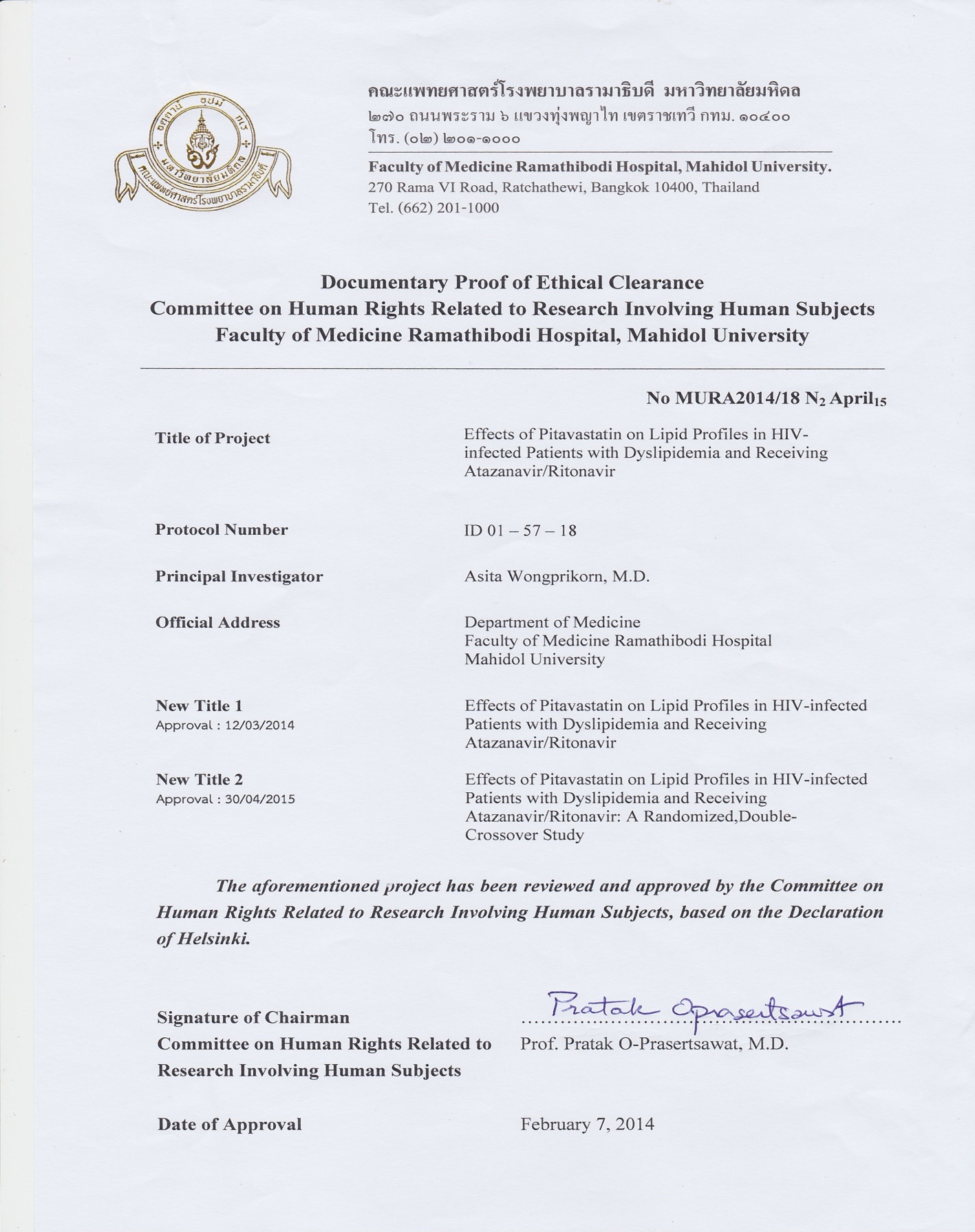

Supplement: S2 Fig — (DOCX) [file pone.0157531.s002.docx]
