# Supplementary material for: Effects of Pitavastatin on Lipid Profiles in HIV-Infected Patients with Dyslipidemia and Receiving Atazanavir/Ritonavir: A Randomized, Double-Blind, Crossover Study
Source: PLoS One. 2016 Jun 15;11(6):e0157531. doi: 10.1371/journal.pone.0157531 (PMC4909195; doi:10.1371/journal.pone.0157531)
Supplement: S4 Fig — (DOC) [file pone.0157531.s004.doc]

**S4 Fig. Full Trial Protocol for Ethical Consideration by the Local Institutional Review Board (IRB) of** **Faculty of Medicine Ramathibodi Hospital, Mahidol University in Thai Language**

**แบบฟอร์มเสนอโครงการฉบับเต็มรูปแบบ สำหรับคณะกรรมการจริยธรรมการวิจัยในคน**

**คณะแพทยศาสตร์โรงพยาบาลรามาธิบดี มหาวิทยาลัยมหิดล**

**1.ชื่อโครงการวิจัย**

(ภาษาไทย) ประสิทธิภาพของยาพิทาวาสเตติน (pitavastatin) ต่อการเปลี่ยนแปลงของระดับไขมันในผู้ติดเชื้อเอชไอวีที่มีระดับไขมันในเลือดสูงขณะได้รับยาอะทาซานาเวียร์/ริโทนาเวียร์ (atazanavir/ritonavir)

(ภาษาอังกฤษ) Effects of Pitavastatin on Lipid Profiles in HIV-infected Patients with Dyslipidemia and Receiving Atazanavir/Ritonavir

***2.* ชื่อหัวหน้าโครงการ**

| ชื่อ | (ไทย) | แพทย์หญิงอสิตา วงศ์ไพรกรณ์ |
| --- | --- | --- |
|  | (อังกฤษ) | Asita Wongprikorn, MD |

คุณวุฒิ แพทยศาสตร์บัณฑิต คณะแพทยศาสตร์ศิริราชพยาบาล มหาวิทยาลัยมหิดล

สถานที่ที่สามารถติดต่อได้ ภาควิชาอายุรศาสตร์ คณะแพทยศาสตร์โรงพยาบาลรามาธิบดี

โทร. 09-1774-5573, 08-7930-7667, 46147

**3. แพทย์ผู้ดูแลโครงการ**

| ชื่อ | (ไทย) | รองศาสตราจารย์แพทย์หญิงศศิโสภิณ เกียรติบูรณกุล |
| --- | --- | --- |
|  | (อังกฤษ) | Assoc. Prof. Sasisopin Kiertiburanakul, MD, MHS |

คุณวุฒิ แพทยศาสตร์บัณฑิต คณะแพทยศาสตร์โรงพยาบาลรามาธิบดี มหาวิทยาลัยมหิดล

Master of Health Science, Johns Hopkins University

**สถานที่ที่สามารถติดต่อได้ สาขาวิชาโรคติดเชื้อ ภาควิชาอายุรศาสตร์ คณะแพทยศาสตร์โรงพยาบาลรามาธิบดีโทร. 0-2201-0033, 08-1808-2223**

| ชื่อ | (ไทย) | แพทย์หญิงอสิตา วงศ์ไพรกรณ์ |
| --- | --- | --- |
|  | (อังกฤษ) | Asita Wongprikorn, MD |

คุณวุฒิ แพทยศาสตร์บัณฑิต คณะแพทยศาสตร์ศิริราชพยาบาล มหาวิทยาลัยมหิดล

สถานที่ที่สามารถติดต่อได้ ภาควิชาอายุรศาสตร์ คณะแพทยศาสตร์โรงพยาบาลรามาธิบดี

โทร. 09-1774-5573, 08-7930-7667, 46147

**4. หลักการและเหตุผล**

โรคหลอดเลือดหัวใจเป็นสาเหตุการตายสำคัญที่พบมากขึ้นเรื่อยๆ ในผู้ติดเชื้อเอชไอวี ในทวีปยุโรปพบอัตราตายจากโรคหลอดเลือดหัวใจเพิ่มขึ้นเรื่อยๆ จากร้อยละ 8 ในปี พ.ศ.2543 เป็นร้อยละ 10 และ 14 ในปี พ.ศ.2548 และ 2553 ตามลำดับ^1^ ทั้งนี้เนื่องมาจากการมีชีวิตที่ยืนยาวขึ้นของผู้ติดเชื้อเอชไอวี เพราะได้รับยาต้านเอชไอวีที่มีประสิทธิภาพมากขึ้นในปัจจุบัน รวมทั้งยาต้านเอชไอวีบางชนิดที่ทำให้ระดับไขมันสูงขึ้นเมื่อรับประทานยาเป็นระยะเวลานาน ซึ่งระดับไขมันที่สูงขึ้นนี้เป็นปัจจัยเสี่ยงที่สำคัญต่อการเกิดโรคหลอดเลือดหัวใจในผู้ติดเชื้อเอชไอวี^2^

ในปัจจุบัน ยาต้านเอชไอวีในกลุ่มโปรตีเอส อินฮิบิเตอร์ (protease inhibitors: PIs) ถูกนำมาใช้ในผู้ติดเชื้อเอชไอวีอย่างแพร่หลายมากขึ้น เนื่องจากยามีคุณสมบัติในการเกิดการดื้อยาค่อนข้างต่ำเมื่อเทียบกับยา กลุ่มนิวคลิโอไซด์ รีเวิร์ส ทรานสคริปเตส อินฮิบิเตอร์ (nucleoside reverse transcriptase inhibitors; NRTIs) และนอนนิวคลิโอไซด์ รีเวิร์ส ทรานสคริปเตส อินฮิบิเตอร์ (non-nucleoside reverse transcriptase inhibitors; NNRTIs)^3^ ในประเทศไทยเองก็มีผู้ติดเชื้อเอชไอวีที่ใช้ยากลุ่มโปรตีเอส อินฮิบิเตอร์เพิ่มมากขึ้น เนื่องมาจากปัญหาการดื้อยาและการมีผลข้างเคียงของยาต้านเอชไอวีกลุ่มอื่นๆ โดยผู้ติดเชื้อเอชไอวีที่ได้รับยากลุ่มโปรตีเอส อินฮิบิเตอร์นี้มักจะพบว่ามีระดับไขมันในเลือดเพิ่มสูงขึ้น^2^ และถึงแม้ว่าผู้ติดเชื้อบางรายจะได้รับยาอะทาซานาเวียร์ (atazanavir) ซึ่งถือเป็นยาต้านเอชไอวีในกลุ่มนี้ที่มีข้อมูลว่าทำให้ระดับไขมันในเลือดเพิ่มสูงน้อยกว่ายาต้านเอชไอวีตัวอื่นๆ แล้วก็ตาม^4^ แต่การรับประทานยาอะทาซานาเวียร์ในผู้ติดเชื้อส่วนใหญ่ต้องใช้ควบคู่ไปกับยาริโทนาเวียร์ (ritonavir) ซึ่งมีคุณสมบัติทำให้ระดับไขมันเพิ่มสูงขึ้นได้^5^ ดังนั้นผู้ติดเชื้อบางรายจึงมีระดับไขมันในเลือดสูงได้แม้ได้รับยาอะทาซานาเวียร์

จากข้อมูลที่ผ่านมา พบว่าผู้ติดเชื้อเอชไอวีที่มีระดับไขมันในเลือดสูงได้ประโยชน์จากการรับประทานยาลดไขมันควบคู่ไปกับการรับประทานยาต้านไวรัสชนิดเดิมมากกว่าการเปลี่ยนยาต้านไวรัสจากกลุ่มที่ทำให้ระดับไขมันในเลือดสูง เช่น จากกลุ่มโปรตีเอส อินฮิบิเตอร์ไปเป็นยาในกลุ่มอื่นที่มีการศึกษาว่าทำให้ระดับไขมันในเลือดเพิ่มสูงขึ้นได้น้อยกว่า เช่น ยาในกลุ่มนอนนิวคลิโอไซด์ รีเวิร์ส ทรานสคริปเตส อินฮิบิเตอร์^6^ ทั้งนี้ยาลดไขมันที่มีใช้ในปัจจุบันมีปัญหาเรื่องการเกิดปฏิกิริยาระหว่างยากับยาต้านเอชไอวีค่อนข้างมาก เนื่องจากยาลดไขมันส่วนใหญ่มีการเมแทบอลิซึม (metabolism) ผ่านทางไซโตโครมพีสี่ห้าศูนย์ (cytochrome P450) เป็นหลัก^7^ เช่นเดียวกับยาต้านเอชไอวีส่วนใหญ่ อนึ่งยาพิทาวาสเตติน (pitavastatin) เป็นยาลดไขมันที่เพิ่งได้รับการรับรองจากองค์การอาหารและยาของประเทศสหรัฐอเมริกาในปี พ.ศ.2552 โดยคุณสมบัติสำคัญของยาที่แตกต่างจากยาลดไขมันตัวอื่นๆ คือ ประสิทธิภาพในการลดระดับไขมัน โดยเฉพาะไขมันแอลดีแอล (Low Density Lipoprotein; LDL) ที่มากกว่ายาลดไขมันตัวอื่น ร่วมกับการมีเมแทบอลิซึมหลักผ่านทางกระบวนการกลูคูโรนิเดชัน (glucuronidation) กลายเป็นสารที่สามารถละลายน้ำและขับออกจากร่างกายผ่านทางปัสสาวะและอุจจาระได้โดยง่าย โดยมีเพียงส่วนน้อยที่มีเมแทบอลิซึมผ่านทางไซโตโครมพีสี่ห้าศูนย์^8^ ดังนั้นปัญหาเรื่องปฏิกิริยาระหว่างยาพิทาวาสเตตินกับยาต้านเอชไอวีจึงเกิดได้น้อยกว่ายาลดไขมันตัวอื่นๆ

ที่ผ่านมา มีเพียงการศึกษาเดียวที่เคยศึกษาเรื่องการลดระดับไขมันโดยใช้ยาพิทาวาสเตตินในผู้ติดเชื้อเอชไอวี ซึ่งผลการศึกษาพบว่ายาพิทาวาสเตตินมีประสิทธิภาพและมีความปลอดภัยที่จะนำมาใช้ในผู้ติดเชื้อเอชไอวีได้^9^ อย่างไรก็ตาม ข้อมูลดังกล่าวเป็นการศึกษาข้อมูลในต่างประเทศ ซึ่งผู้ติดเชื้อในประเทศไทย มีความแตกต่างทั้งด้านข้อมูลพื้นฐานและกระบวนการเมแทบอลิซึมของยาเป็นสำคัญ ดังนั้นผู้วิจัยจึงได้จัดทำโครงการวิจัยนี้ขึ้นมาเพื่อศึกษาถึงประสิทธิภาพและความปลอดภัยของยาพิทาวาสเตตินในผู้ป่วยเอชไอวีที่รับประยาอะทาซานาเวียร์/ริโทนาเวียร์ในประเทศไทย ซึ่งหากผลการศึกษาออกมาเป็นที่น่าพอใจ ผู้ติดเชื้อเอชไอวีจะได้มียาลดไขมันที่ปลอดภัยและราคาไม่แพงเป็นอีกหนึ่งทางเลือกในการควบคุมระดับไขมันต่อไป

**5. วัตถุประสงค์**

5.1 วัตถุประสงค์หลัก เพื่อศึกษาประสิทธิภาพของยาพิทาวาสเตตินในผู้ติดเชื้อเอชไอวีที่มีระดับไขมันในเลือดสูงขณะได้รับยาอะทาซานาเวียร์/ริโทนาเวียร์

5.2 วัตถุประสงค์รอง เพื่อศึกษาความปลอดภัยของการใช้ยาพิทาวาสเตตินในผู้ติดเชื้อเอชไอวี

**6. วิธีการวิจัยและแบบแผนการวิจัย**

ใช้วิธีการศึกษาเชิงทดลองแบบปกปิดทั้งสองด้าน (randomized, double-blinded, controlled trial) ด้วยวิธีศึกษาแบบไขว้ (crossover study) ในผู้ติดเชื้อเอชไอวีที่มารับการตรวจที่คลินิกโรคติดเชื้อ แผนกผู้ป่วยนอกที่โรงพยาบาลรามาธิบดีในช่วงระยะเวลาตั้งแต่เดือนมีนาคมจนถึงธันวาคม 2557

*วิธีการดำเนินการวิจัย*

1. ผู้ติดเชื้อเอชไอวีที่รับประทานยาอะทาซานาเวียร์/ริโทนาเวียร์ที่มารับการรักษาที่แผนกผู้ป่วยนอกของสาขาวิชาโรคติดเชื้อโรงพยาบาลรามาธิบดีจะได้รับการตรวจโดยแพทย์
2. ซักประวัติผู้ติดเชื้อ ถ้าพบว่าเข้ากับข้อกำหนด แพทย์ผู้ตรวจจะให้ข้อมูลและคำอธิบายเกี่ยวกับการศึกษากับผู้ติดเชื้อ
3. เก็บข้อมูลพื้นฐานที่จำเป็นของผู้ติดเชื้อที่ยินยอมเข้าร่วมการศึกษา
4. ผู้ติดเชื้อจะได้รับการตรวจระดับไขมัน ระดับน้ำตาล การทำงานของตับและไตก่อนเข้าร่วมการศึกษา
5. ผู้ติดเชื้อจะได้รับการแบ่งกลุ่มแบบสุ่มในการเริ่มยาหลอก (placebo) หรือยาพิทาวาสเตตินก่อน โดยการแบ่งกลุ่มนี้ทำโดยคอมพิวเตอร์ และผลการสุ่มจะถูกใส่ซองจดหมายปิดผนึกเรียงลำดับ โดยที่ทั้งแพทย์และผู้ติดเชื้อไม่ทราบว่าผู้ติดเชื้ออยู่ในกลุ่มการทดลองใด
6. เมื่อผู้ติดเชื้อแต่ละกลุ่มได้รับยาตามที่กำหนดแล้ว จะได้รับการนัดหมายมาตรวจเลือดและพบแพทย์เพื่อตรวจร่างกายตามระยะเวลาที่กำหนด
7. ประเมินผลที่ได้นำมาวิเคราะห์เพื่อพิจารณาประสิทธิภาพและความปลอดภัยของการใช้ยาพิทาวาสเตติน เพื่อลดระดับไขมันในผู้ติดเชื้อเอชไอวีต่อไป

*การรวบรวมข้อมูลทางการแพทย์*

ผู้วิจัยจะเก็บบันทึกข้อมูลทางการแพทย์ทั้งหมดที่เป็นข้อมูลต้นฉบับในแบบฟอร์มบันทึกข้อมูล (case report form; CRF) ซึ่งได้รับการออกแบบขึ้นมาเพื่อใช้ในโครงการนี้โดยเฉพาะ โดยจะเก็บรวบรวมข้อมูลด้วยการสัมภาษณ์ผู้ป่วย เวชระเบียน รายงานจากห้องปฏิบัติการ ข้อมูลการวิจัยจะได้รับการเก็บรวบรวมที่สถานพยาบาลและลงบันทึกในแบบฟอร์ม

**7. Protocol flow chart**

ผู้ติดเชื้อเอชไอวีที่รับประทานยาอะทาซานาเวียร์/ริโทนาเวียร์และมีระดับไขมันในเลือดสูง 24 ราย

เก็บข้อมูลพื้นฐานและเจาะเลือดตรวจระดับไขมัน [total cholesterol (TC), Triglyceride (TG), LDL, HDL] ระดับน้ำตาล (FBS) การทำงานของตับ (AST, ALT) และไต (creatinine) และค่าการอักเสบของผู้ติดเชื้อก่อนเข้าร่วมการวิจัย

2 สัปดาห์

หยุดยาหลอก

หยุดยาพิทาวาสเตติน

หลังครบระยะเวลา 3 เดือน ให้ทั้งสองกลุ่มหยุดยา แล้วประเมินผลการวิจัย

*นัดมาตรวจติดตามการรักษาทุก 1 เดือน

3 เดือน

เปลี่ยนมารับประทานยาพิทาวาสเตติน

เปลี่ยนมารับประทานยาหลอก

3 เดือน

**กลุ่มที่ 1**

เริ่มรับประทานยาหลอกก่อน

**กลุ่มที่ 2**

เริ่มรับประทานยาพิทาวาสเตตินก่อน

*นัดมาตรวจติดตามการรักษาทุก 1 เดือน

แบ่งผู้ติดเชื้อเป็น 2 กลุ่มโดยใช้คอมพิวเตอร์สุ่ม โดยที่ทั้งแพทย์และผู้ติดเชื้อไม่ทราบว่าตนเองอยู่ในกลุ่มการศึกษาใด

หมายเหตุ * ซักประวัติและตรวจร่างกายประเมินความสม่ำเสมอและผลข้างเคียงจากการรับประทานยา

เจาะเลือดดูระดับไขมัน (TC, TG, LDL, HDL) และการทำงานของตับ (AST, ALT) ทุกครั้ง

ตรวจระดับยาอะทาซานาเวียร์ ระดับยาพิทาวาสเตติน ค่าการอักเสบและระดับครีเอทีน ไคเนส

(Creatine kinase) ที่ 3 และ 6 เดือน

**8. จำนวนผู้เข้าร่วมการวิจัย เกณฑ์คัดเข้าและเกณฑ์คัดออก**

จำนวนประชากรที่ทำการศึกษา 24 ราย (ดูวิธีการคำนวณที่ภาคผนวก)

8.1 เกณฑ์การเข้าร่วมโครงการ (inclusion criteria)

• ยินดีเข้าร่วมโครงการโดยการลงนาม

- มีผลการตรวจยืนยันการติดเชื้อเอชไอวี โดยมีเชื้อไวรัสเอชไอวี หรือผลการตรวจใดๆ ที่ได้รับการรับรองโดยวิธีทดสอบแบบ ELISA และได้รับการยืนยันโดยวิธีอื่นต่างๆ ไม่จำกัดเพียงการตรวจเอชไอวีแบบรวดเร็ว, Westen Blot, การตรวจเฉพาะเชื้อเอชไอวี, แอนติเจนของไวรัสเอชไอวี หรือการตรวจปริมาณเชื้อเอชไอวีในเวลาใดได้ตลอดเวลา
- อายุมากกว่าหรือเท่ากับ 18 ปี
- ได้รับยาต้านเอชไอวีที่มียาอะทาซานาเวียร์ขนาด 300 มิลลิกรัม ร่วมกับยาริโทนาเวียร์ขนาด 100 มิลลิกรัม ร่วมกับยาต้านเอชไอวีตัวอื่นโดยไม่มีการเปลี่ยนแปลงสูตรยาดังกล่าวภายในระยะเวลาอย่างน้อย 3 เดือนก่อนเข้าร่วมการศึกษาวิจัยในครั้งนี้
- มีระดับไขมันในเลือดสูง โดยตรวจพบระดับโคเลสเตอรอลมากกว่าหรือเท่ากับ 200 แต่ไม่เกิน 500 มิลลิกรัม/เดซิลิตร หรือมีระดับไขมันแอลดีแอล (Low Density Lipoprotein; LDL) มากกว่าหรือเท่ากับ 130 แต่ไม่เกิน 400 มิลลิกรัม/เดซิลิตร
- ไม่ได้รับยาลดไขมันใดๆ หรือได้รับการหยุดยาลดไขมันที่เคยรับประทานอยู่มาเป็นระยะเวลาอย่างน้อย 1 เดือนก่อนหน้าที่จะเข้าร่วมการศึกษาวิจัยในครั้งนี้

8.2 เกณฑ์การคัดออกจากโครงการวิจัย (exclusion criteria)

- มีประวัติแพ้ยาหรือส่วนประกอบของยาพิทาวาสเตติน
- มีประวัติเป็นโรคกล้ามเนื้อหัวใจตายเฉียบพลันหรือโรคหลอดเลือดสมองตีบภายในระยะเวลา 1 เดือนก่อนเข้าร่วมการวิจัย ซึ่งอาจเกิดอันตรายหากหยุดยาลดไขมันก่อนเข้าร่วมการวิจัย
- มีตับอักเสบ ซึ่งตรวจพบจากการเจาะเลือดก่อนเข้าร่วมการวิจัย โดยมีระดับ aspartate amino-

transferase (AST) และ alanine aminotransaminase (ALT) สูงมากกว่าหรือเท่ากับ 5 เท่าในผู้ที่ไม่มีอาการของตับอักเสบหรือ 3 เท่าในผู้ที่มีอาการผิดปกติร่วมด้วย

- เป็นสตรีที่อยู่ในระยะตั้งครรภ์หรือให้นมบุตร
- รับประทานยาไซโคลสปอริน (cyclosporine) ซึ่งมีปฏิกิริยาระหว่างยาที่รุนแรงกับยาพิทาวาสเตติน
- ปฏิเสธหรือขอถอนตัวออกจากการวิจัย

**9. ระยะเวลาในการศึกษา**

ระยะเวลาในการดำเนินการวิจัยประมาณ 10 เดือน ตั้งแต่เดือนมีนาคม ถึงธันวาคม พ.ศ. 2557

**10. ความเสี่ยงหรือความไม่สบายที่คาดว่าจะเกิดขึ้นกับผู้เข้าร่วมการวิจัย**

• ผู้เข้าร่วมการวิจัยจะต้องเจาะเลือดเพื่อการตรวจตัวอย่างเลือด การเจาะเลือดจากแขนของท่านอาจทำให้ท่านเจ็บ เกิดจ้ำเลือด อาการคล้ายเป็นลม และการติดเชื้อซึ่งพบน้อยมาก โดยตลอดทั้งการศึกษานี้ท่านจะถูกเจาะเลือดทั้งหมด 8 ครั้ง ครั้งละประมาณ 10-15 มิลลิลิตร (หรือประมาณ 2-3 ช้อนโต๊ะ)

**11. ประโยชน์ที่คาดว่าจะได้รับ**

• การเข้าร่วมโครงการวิจัยนี้อาจเกิดประโยชน์ต่ออาสาสมัครและผู้ติดเชื้อเอชไอวีรายอื่นๆ หากผลการศึกษาพบว่ายาลดไขมันพิทาวาสเตตินมีประสิทธิภาพดีและปลอดภัยในการใช้ลดระดับไขมันในผู้ติดเชื้อเอชไอวี

**12. ข้อพิจารณาด้านจริยธรรม**

- การทำวิจัยครั้งนี้ดำเนินการตามหลักจริยธรรมตามคำประกาศเฮลซิงกิ
- การเข้าร่วมโครงการเป็นไปโดยความสมัครใจของอาสาสมัคร
- อาสาสมัครสามารถปฏิเสธที่จะเข้าร่วมโครงการวิจัยนี้ โดยที่อาสาสมัครจะไม่เสียผลประโยชน์ใดๆ ในการรักษาตามปกติ
- ผู้วิจัยจะปกปิดข้อมูลส่วนตัวเกี่ยวกับสุขภาพของอาสาสมัครและป้องกันไม่ให้มีการใช้ข้อมูลในทางที่ผิด

**13. ข้อชดเชยแก่ผู้เข้าร่วมการวิจัย**

ผู้เข้าร่วมโครงการวิจัยจะได้รับการตรวจทางห้องปฏิบัติการดังกล่าวข้างต้นรวมทั้งยาพิทาวาสเตตินโดยไม่คิดมูลค่าและได้รับค่าเดินทางครั้งละ 300 บาท เพื่อมาเจาะตรวจเลือดตามระยะเวลาที่กำหนด พร้อมกับการได้รับคำแนะนำเกี่ยวกับการลดระดับไขมัน ซึ่งมีความสำคัญในการช่วยลดปัจจัยเสี่ยงต่อการเกิดโรคหลอดเลือดหัวใจ

**14. แหล่งทุนวิจัย**

ผู้วิจัยได้รับการสนับสนุนจากบริษัทไบโอฟาร์ม เคมิคัลส์ จำกัด เฉพาะในส่วนของยาพิทาวาสเตตินและยาหลอกที่จำเป็นต้องผลิตออกมาให้มีขนาด รูปร่าง สี และกลิ่นใกล้เคียงกันมากที่สุด ส่วนค่าใช้จ่ายในการเจาะเลือดตรวจทางห้องปฏิบัติการและค่าใช้จ่ายเพื่อสนับสนุนการเดินทางของผู้ป่วยให้มาพบแพทย์ตามนัดทุกครั้ง ผู้วิจัยได้ขอทุนสนับสนุนจากทางคณะแพทยศาสตร์โรงพยาบาลรามาธิบดี มหาวิทยาลัยมหิดล

**15. เอกสารชี้แจงข้อมูลและคำแนะนำแก่ผู้เข้าร่วมโครงการ (patient /participant information sheet)**

เอกสารประกอบ 1

**16. หนังสือยินยอมโดยได้รับการบอกกล่าวและเต็มใจ (inform consent form)**

เอกสารประกอบ 2

ลงนาม .........................................................

แพทย์หญิงอสิตา วงศ์ไพรกรณ์

(หัวหน้าโครงการ)

ลงนาม .........................................................

รองศาสตราจารย์แพทย์หญิงศศิโสภิณ เกียรติบูรณกุล

(อาจารย์ที่ปรึกษา)

**เอกสารอ้างอิง**

Henard S, Roussillon C, Bonnet F, et al. Cardiovascular-related deaths in HIV positive patients between 2000 and 2010: Agence Nationale de Recherche sur le Sida EN20 Mortalite 2010 Survey. 20^th^ Conference on Retroviruses and Opportunistic Infections. March 3-6, 2013; Atlanta. Abstract 1048.

Estrada V and Portilla J. Dyslipidemia related to antiretroviral therapy. AIDS Rev 2011;13:49-56.

Arora D, Dixit NM. Timing the emergence of resistance to anti-HIV drugs with large genetic barriers. PLoS Comput Biol 2009;5(3): e1000305.

Luwdgren JD, Battegay M, Behrens G, Wit SD, Guaraldi G, Katlama C, et al. European AIDS Clinical Society (EACS) guidelines on the prevention and management of metabolic diseases in HIV. HIV Med 2008;9:72-81.

Dube PM, Stein JH, Aberg JA, Fichtenbaum CJ, Gerber JG, Tashima KT, et al. Guidelines for the evaluation and management of dyslipidemia in Human Immunodeficiency Virus (HIV)-infected adults receiving antiretroviral therapy: Recommendations of the HIV Medicine Association of the Infectious Disease Society of America and the Adult AIDS Clinical Trials Group. Clin Infect Dis 2003;37:613-27.

Calza L, Manfredi R, Colangeli V, Tampellini L, Sebastiani T, Pocaterra D, et al. Substitution of nevirapine or efavirenz for protease inhibitor versus lipid-lowering therapy for the management of dyslipidemia. AIDS 2005;19:1051-8.

Neuvonen DJ, Niemi M, Backman JT. Drug interactions with lipid-lowering drugs mechanisms and clinical relevance. Clin Pharmacol Ther 2006;80:565-81.

Full prescribing information of pitavastatin, reference ID: 3090715.

Sponsellar CA, Morgan RE, Campbell SE, Kryzhanovski VA, Kartman CE, Aberg JA, et al. Pitavastatin 4 mg provides superior LDL-C reduction versus pravastatin 40 mg over 12 weeks in HIV-infected adults with dyslipidemia, the INTREPID trial. 20^th^ Conference on Retroviruses and Opportunistic Infections. March 3, 2013; Atlanta. Abstract 139.

**ภาคผนวก**

*วิธีคำนวณขนาดตัวอย่าง (Sample size)*

ใช้โปรแกรมสำเร็จรูปคำนวณขนาดประชากร power and sample size calculations version 3.0.43 โดยเลือกรูปแบบการศึกษาแบบเป็นคู่ทีเทสต์ (paired design T-test) และกำหนดให้อำนาจของการทดสอบ (power) เท่ากับร้อยละ 80 ค่าความคลาดเคลื่อนที่ยอมรับได้ (type I error) ไม่เกินร้อยละ 5 ความแตกต่างของค่าเฉลี่ยของข้อมูลแต่ละคู่ (difference of means) และค่าเบี่ยงเบนมาตรฐาน (standard deviation) เท่ากับ 20 โดยอ้างอิงมาจากการศึกษาก่อนหน้านี้^9^

หลังจากแทนค่าดังกล่าวในโปรแกรมสำเร็จรูปจะได้จำนวนประชากรทั้งหมด 10 คู่ เท่ากับ 20 คน และคิดเพิ่มร้อยละ 20 ในกรณีที่ผู้ป่วยออกจากการศึกษาหรือไม่สามารถติดตามผู้ป่วยได้ อีก 4 คน รวมเป็นจำนวนประชากรทั้งหมด 24 คน
