# Supplementary material for: Effects of Pitavastatin on Lipid Profiles in HIV-Infected Patients with Dyslipidemia and Receiving Atazanavir/Ritonavir: A Randomized, Double-Blind, Crossover Study
Source: PLoS One. 2016 Jun 15;11(6):e0157531. doi: 10.1371/journal.pone.0157531 (PMC4909195; doi:10.1371/journal.pone.0157531)
Supplement: S5 Fig — (DOCX) [file pone.0157531.s005.docx]

**S5 Fig. Case Record Form**

**Randomization form**

Participant ID (PID): |_|_|

This participant was randomized on |_|_|-|_|_|-|_|_| to the following arm.

*dd*  *mm* *yy*

□ Arm A

□ Arm B

**Demographic data**

Name: __________________________________________ Age |_|_| years

Gender: □ Female □ Male

PID: |_|_| HN: |_|_|_|_|_|_|_|

Telephone number: _______________________________________________

Address _________________________________________________________

**Screening visit**

PID: |_|_|

Date of visit: |_|_|-|_|_|-|_|_|

*dd*  *mm* *yy*

Written informed consent is obtained □ Yes Date: |_|_|-|_|_|-|_|_|

□ No *dd*  *mm* *yy*

**1. Demographic data**

Ethnic group: 1□ Thai 2□ Other specify: _______________

Marital status: 1□ Single 2□ Married

3□ Widow/ Seperated/ Divorced

HIV risk factor: 1□ Heterosexual 2□ Homosexual

3□ IV drug use

4□ Other/ Unknown specify: _________________

CDC HIV classification: 1□ A 2□ B 3□ C

**2. HIV-related illness history**

| **Diagnosis** | **Start date**  *(dd mm yy)* | **Stop date**  (tick if □ ongoing)  *(dd mm yy)* |
| --- | --- | --- |
|  | __ /__ /__ | __ /__ /__ □ |
|  | __ /__ /__ | __ /__ /__ □ |
|  | __ /__ /__ | __ /__ /__ □ |
|  | __ /__ /__ | __ /__ /__ □ |
|  | __ /__ /__ | __ /__ /__ □ |

**3. Medical history**

| **Diagnosis** | **Start date**  *(dd mm yy)* | **Stop date**  (tick if □ ongoing)  *(dd mm yy)* |
| --- | --- | --- |
|  | __ /__ /__ | __ /__ /__ □ |
|  | __ /__ /__ | __ /__ /__ □ |
|  | __ /__ /__ | __ /__ /__ □ |
|  | __ /__ /__ | __ /__ /__ □ |
|  | __ /__ /__ | __ /__ /__ □ |
|  | __ /__ /__ | __ /__ /__ □ |
|  | __ /__ /__ | __ /__ /__ □ |
|  | __ /__ /__ | __ /__ /__ □ |
|  | __ /__ /__ | __ /__ /__ □ |
|  | __ /__ /__ | __ /__ /__ □ |

**4. Medication history**

| **Medication Name** | **Start date**  *(dd mm yy)* | **Stop date**  (tick if □ ongoing)  *(dd mm yy)* |
| --- | --- | --- |
|  | __ /__ /__ | __ /__ /__ □ |
|  | __ /__ /__ | __ /__ /__ □ |
|  | __ /__ /__ | __ /__ /__ □ |
|  | __ /__ /__ | __ /__ /__ □ |
|  | __ /__ /__ | __ /__ /__ □ |
|  | __ /__ /__ | __ /__ /__ □ |
|  | __ /__ /__ | __ /__ /__ □ |
|  | __ /__ /__ | __ /__ /__ □ |
|  | __ /__ /__ | __ /__ /__ □ |
|  | __ /__ /__ | __ /__ /__ □ |
|  | __ /__ /__ | __ /__ /__ □ |

**5. Antiretroviral therapy history**

| **ARV Name** | **Start date**  *(dd mm yy)* | **Stop date**  (tick if □ ongoing)  *(dd mm yy)* | **If stop ART, indicate reason**  *(can tick more than one)* |
| --- | --- | --- | --- |
|  | __ /__ /__ | __ /__ /__ □ | 1□ 2□ 3□  4□ 5□ 6□  7□ _________ |
|  | __ /__ /__ | __ /__ /__ □ | 1□ 2□ 3□  4□ 5□ 6□  7□ _________ |
|  | __ /__ /__ | __ /__ /__ □ | 1□ 2□ 3□  4□ 5□ 6□  7□ _________ |
|  | __ /__ /__ | __ /__ /__ □ | 1□ 2□ 3□  4□ 5□ 6□  7□ _________ |
|  | __ /__ /__ | __ /__ /__ □ | 1□ 2□ 3□  4□ 5□ 6□  7□ _________ |
|  | __ /__ /__ | __ /__ /__ □ | 1□ 2□ 3□  4□ 5□ 6□  7□ _________ |

**Note:** 1: Clinical failure, 2: Immunological failure, 3: Virological failure,

4: Socioeconomic problem, 5: Adherence

6: Toxicity, 7: Other (specify) ___________________

**6. CD4 cell counts and HIV viral load most recent date**

Date: |_|_| |_|_| |_|_| CD4 |_|_| % |_|_|_|_| cells/ mm^3^

Date: |_|_| |_|_| |_|_| HIV-RNA >□ <□ =□ |_|_|_|_|_|_| copies/ ml

**7. Vital signs**

Body weight (BW): |_|_|_|.|_| kg

Height: |_|_|_|.|_| cm

Body Mass Index (BMI): |_|_|.|_| kg/m^2^

**8. Screening lab**

1) Fasting blood sugar (FBS) |_|_|_|_| mg/dL

2) Creatinine (Cr) |_|_|_|_| mg/dL

3) Aspartate aminotransferase (AST) |_|_|_|_| U/L

Alanine aminotransaminase (ALT) |_|_|_|_| U/L

4) Lipid profiles

Total cholesterol (TC) |_|_|_|_| mg/dL

Triglyceride (TG) |_|_|_|_| mg/dL

Low Density Lipoprotein (LDL) |_|_|_|_| mg/dL

High Density Lipoprotein (HDL) |_|_|_|_| mg/dL

**9. Cardiovascular (CV) risk factors**

1. Smoking □ *Current smoking*

□ Never smoking

□ Smoking cessation No. __ pack/ day since _____

2. Hypertension (HT) □ *Systolic Blood Pressure (SBP) ≥140 mmHg*

□ *on antihypertensive drug*

□ No HT

3. HDL □ *<40 mg/dL*  □ >40 mg/dL

4. Family history of premature coronary heart disease (CHD)

□ *Male first-degree relative <55 years old*

□ *Female first-degree relative <65 years old*

□ No family history of premature CHD

5. Age □ *Male >45 years old* □ *Female >55 years old*

□ Male ≤45 years old □ Female ≤55 years old

1*□* <2 CV risk factors

2*□ ≥2 CV risk factors: 10-year-risk 1□ <10% 2□ 10-20% 3□ <20%*

**10. Physical examination** (Tick box if normal, describe if abnormal)

□ General appearance ______________ □ Skin _________________________

□ Eyes ___________________________ □ Ear, nose, throat _______________

□ Cardiovascular ___________________ □ Respiratory __________________

□ Gastrointestinal __________________ □ Neurological _________________

□ Musculoskeletal __________________ □ Others: _____________________

**11. Eligibility criteria**

*1. Inclusion criteria* (All answers must be ‘Yes’.) *Yes* No

1) HIV-infected adults age ≥18 years □ □

2) Currently on atazanavir (ATV) 300 mg and □ □

ritonavir (RTV) 100 mg with stable other

antiretroviral drugs (ARV) at least 3 months

prior screening visit

3) Dyslipidemia defined by □ □

3.1) TC: range 200-500 mg/dL and/or

3.2) LDL: range 130-400 mg/dL

4) Not currently on lipid-lowering agent or □ □

minimum 4 weeks washout previous

lipid-lowering agent prior screening visit

*2. Exclusion criteria* (All answers must be ‘No’) Yes *No*

1) Known history of hypersensitivity to any □ □

component of pitavastatin

2) History of myocardial infarction (MI) or □ □

ischemic stroke within 1 month prior

screening visit

3) AST and ALT ≥5 times of upper normal limit (UNL) in □ □

asymptomatic or ≥3 times of UNL in symptomatic

patient

4) Pregnancy and lactation □ □

5) Currently on cyclosporine with severe drug □ □

interaction with pitavastatin

Follow up date: |_|_| |_|_| |_|_|

*dd mm yy*

Week 4

PID: |_|_|

Date of visit: |_|_|-|_|_|-|_|_|

*dd*  *mm* *yy*

1. **Medical history**

**___________________________________________________________________________________________________________________________________________________________________________________________________________________________________________________________________________________________________________________________________**

1. **Vital signs**

Body weight (BW): |_|_|_|.|_| kg

Height: |_|_|_|.|_| cm

Body Mass Index (BMI): |_|_|.|_| kg/m^2^

1. **HIV-related illnesses**

| **Diagnosis** | **Start date**  *(dd mm yy)* | **Stop date**  (tick if □ ongoing)  *(dd mm yy)* |
| --- | --- | --- |
|  | __ /__ /__ | __ /__ /__ □ |
|  | __ /__ /__ | __ /__ /__ □ |
|  | __ /__ /__ | __ /__ /__ □ |
|  | __ /__ /__ | __ /__ /__ □ |
|  | __ /__ /__ | __ /__ /__ □ |

1. **Adverse events (AEs)**

1□ Yes (If yes, please specify below) 0□ No

| **Description** | **Start date**  *(dd mm yy)* | **Stop date**  (tick if □ ongoing)  *(dd mm yy)* | **Severity** |
| --- | --- | --- | --- |
|  | __ /__ /__ | __ /__ /__ □ |  |
|  | __ /__ /__ | __ /__ /__ □ |  |
|  | __ /__ /__ | __ /__ /__ □ |  |
|  | __ /__ /__ | __ /__ /__ □ |  |
|  | __ /__ /__ | __ /__ /__ □ |  |

**Note:** 1=Mild, 2=Moderate, 3=Severe, 4=Life-threatening

1. **Concomitant medication**: new medication or dose change

1□ Yes (If yes, please specify below) 0□ No

| **Medication Name** | **Start date**  *(dd mm yy)* | **Stop date**  (tick if □ ongoing)  *(dd mm yy)* |
| --- | --- | --- |
|  | __ /__ /__ | __ /__ /__ □ |
|  | __ /__ /__ | __ /__ /__ □ |
|  | __ /__ /__ | __ /__ /__ □ |
|  | __ /__ /__ | __ /__ /__ □ |
|  | __ /__ /__ | __ /__ /__ □ |

**6. Physical examination** (Tick box if normal, describe if abnormal)

□ General appearance ______________ □ Skin _________________________

□ Eyes ___________________________ □ Ear, nose, throat _______________

□ Cardiovascular ___________________ □ Respiratory __________________

□ Gastrointestinal __________________ □ Neurological _________________

□ Musculoskeletal __________________ □ Others: _____________________

1. **Pills count**: |_|_| tabs
2. **Laboratory monitoring**

1) Aspartate aminotransferase (AST) |_|_|_|_| U/L

Alanine aminotransaminase (ALT) |_|_|_|_| U/L

2) Lipid profiles

Total cholesterol (TC) |_|_|_|_| mg/dL

Triglyceride (TG) |_|_|_|_| mg/dL

Low Density Lipoprotein (LDL) |_|_|_|_| mg/dL

High Density Lipoprotein (HDL) |_|_|_|_| mg/dL

3) Creatinine kinase (If complaint) |_|_|_|_| U/L

1. **Note**

**________________________________________________________________________________________________________________________________**

Follow up date: |_|_| |_|_| |_|_|

*dd mm yy*

Week 8

PID: |_|_|

Date of visit: |_|_|-|_|_|-|_|_|

*dd*  *mm* *yy*

1. **Medical history**

**___________________________________________________________________________________________________________________________________________________________________________________________________________________________________________________________________________________________________________________________________**

1. **Vital signs**

Body weight (BW): |_|_|_|.|_| kg

Height: |_|_|_|.|_| cm

Body Mass Index (BMI): |_|_|.|_| kg/m^2^

1. **HIV-related illnesses**

| **Diagnosis** | **Start date**  *(dd mm yy)* | **Stop date**  (tick if □ ongoing)  *(dd mm yy)* |
| --- | --- | --- |
|  | __ /__ /__ | __ /__ /__ □ |
|  | __ /__ /__ | __ /__ /__ □ |
|  | __ /__ /__ | __ /__ /__ □ |
|  | __ /__ /__ | __ /__ /__ □ |
|  | __ /__ /__ | __ /__ /__ □ |

1. **Adverse events (AEs)**

1□ Yes (If yes, please specify below) 0□ No

| **Description** | **Start date**  *(dd mm yy)* | **Stop date**  (tick if □ ongoing)  *(dd mm yy)* | **Severity** |
| --- | --- | --- | --- |
|  | __ /__ /__ | __ /__ /__ □ |  |
|  | __ /__ /__ | __ /__ /__ □ |  |
|  | __ /__ /__ | __ /__ /__ □ |  |
|  | __ /__ /__ | __ /__ /__ □ |  |
|  | __ /__ /__ | __ /__ /__ □ |  |

**Note:** 1=Mild, 2=Moderate, 3=Severe, 4=Life-threatening

1. **Concomitant medication**: new medication or dose change

1□ Yes (If yes, please specify below) 0□ No

| **Medication Name** | **Start date**  *(dd mm yy)* | **Stop date**  (tick if □ ongoing)  *(dd mm yy)* |
| --- | --- | --- |
|  | __ /__ /__ | __ /__ /__ □ |
|  | __ /__ /__ | __ /__ /__ □ |
|  | __ /__ /__ | __ /__ /__ □ |
|  | __ /__ /__ | __ /__ /__ □ |
|  | __ /__ /__ | __ /__ /__ □ |

**6. Physical examination** (Tick box if normal, describe if abnormal)

□ General appearance ______________ □ Skin _________________________

□ Eyes ___________________________ □ Ear, nose, throat _______________

□ Cardiovascular ___________________ □ Respiratory __________________

□ Gastrointestinal __________________ □ Neurological _________________

□ Musculoskeletal __________________ □ Others: _____________________

**7. Pills count**: |_|_| tabs

1. **Laboratory monitoring**

1) Aspartate aminotransferase (AST) |_|_|_|_| U/L

Alanine aminotransaminase (ALT) |_|_|_|_| U/L

2) Lipid profiles

Total cholesterol (TC) |_|_|_|_| mg/dL

Triglyceride (TG) |_|_|_|_| mg/dL

Low Density Lipoprotein (LDL) |_|_|_|_| mg/dL

High Density Lipoprotein (HDL) |_|_|_|_| mg/dL

3) Creatinine kinase (If complaint) |_|_|_|_| U/L

1. **Note**

**________________________________________________________________________________________________________________________________**

Follow up date: |_|_| |_|_| |_|_|

*dd mm yy*

Week 12

PID: |_|_|

Date of visit: |_|_|-|_|_|-|_|_|

*dd*  *mm* *yy*

1. **Medical history**

**___________________________________________________________________________________________________________________________________________________________________________________________________________________________________________________________________________________________________________________________________**

1. **Vital signs**

Body weight (BW): |_|_|_|.|_| kg

Height: |_|_|_|.|_| cm

Body Mass Index (BMI): |_|_|.|_| kg/m^2^

1. **HIV-related illnesses**

| **Diagnosis** | **Start date**  *(dd mm yy)* | **Stop date**  (tick if □ ongoing)  *(dd mm yy)* |
| --- | --- | --- |
|  | __ /__ /__ | __ /__ /__ □ |
|  | __ /__ /__ | __ /__ /__ □ |
|  | __ /__ /__ | __ /__ /__ □ |
|  | __ /__ /__ | __ /__ /__ □ |
|  | __ /__ /__ | __ /__ /__ □ |

1. **Adverse events (AEs)**

1□ Yes (If yes, please specify below) 0□ No

| **Description** | **Start date**  *(dd mm yy)* | **Stop date**  (tick if □ ongoing)  *(dd mm yy)* | **Severity** |
| --- | --- | --- | --- |
|  | __ /__ /__ | __ /__ /__ □ |  |
|  | __ /__ /__ | __ /__ /__ □ |  |
|  | __ /__ /__ | __ /__ /__ □ |  |
|  | __ /__ /__ | __ /__ /__ □ |  |
|  | __ /__ /__ | __ /__ /__ □ |  |

**Note:** 1=Mild, 2=Moderate, 3=Severe, 4=Life-threatening

1. **Concomitant medication**: new medication or dose change

1□ Yes (If yes, please specify below) 0□ No

| **Medication Name** | **Start date**  *(dd mm yy)* | **Stop date**  (tick if □ ongoing)  *(dd mm yy)* |
| --- | --- | --- |
|  | __ /__ /__ | __ /__ /__ □ |
|  | __ /__ /__ | __ /__ /__ □ |
|  | __ /__ /__ | __ /__ /__ □ |
|  | __ /__ /__ | __ /__ /__ □ |
|  | __ /__ /__ | __ /__ /__ □ |

**6. Physical examination** (Tick box if normal, describe if abnormal)

□ General appearance ______________ □ Skin _________________________

□ Eyes ___________________________ □ Ear, nose, throat _______________

□ Cardiovascular ___________________ □ Respiratory __________________

□ Gastrointestinal __________________ □ Neurological _________________

□ Musculoskeletal __________________ □ Others: _____________________

1. **Pills count**: |_|_| tabs
2. **Laboratory monitoring**

1) Aspartate aminotransferase (AST) |_|_|_|_| U/L

Alanine aminotransaminase (ALT) |_|_|_|_| U/L

2) Lipid profiles

Total cholesterol (TC) |_|_|_|_| mg/dL

Triglyceride (TG) |_|_|_|_| mg/dL

Low Density Lipoprotein (LDL) |_|_|_|_| mg/dL

High Density Lipoprotein (HDL) |_|_|_|_| mg/dL

3) Creatinine kinase |_|_|_|_| U/L

4) ATV level |_|_|_|.|_| mcg/L

1. **Note**

**________________________________________________________________________________________________________________________________**

Follow up date: |_|_| |_|_| |_|_|

*dd mm yy*

Week 14

PID: |_|_|

Date of visit: |_|_|-|_|_|-|_|_|

*dd*  *mm* *yy*

1. **Medical history**

**___________________________________________________________________________________________________________________________________________________________________________________________________________________________________________________________________________________________________________________________________**

1. **Vital signs**

Body weight (BW): |_|_|_|.|_| kg

Height: |_|_|_|.|_| cm

Body Mass Index (BMI): |_|_|.|_| kg/m^2^

1. **HIV-related illnesses**

| **Diagnosis** | **Start date**  *(dd mm yy)* | **Stop date**  (tick if □ ongoing)  *(dd mm yy)* |
| --- | --- | --- |
|  | __ /__ /__ | __ /__ /__ □ |
|  | __ /__ /__ | __ /__ /__ □ |
|  | __ /__ /__ | __ /__ /__ □ |
|  | __ /__ /__ | __ /__ /__ □ |
|  | __ /__ /__ | __ /__ /__ □ |

1. **Adverse events (AEs)**

1□ Yes (If yes, please specify below) 0□ No

| **Description** | **Start date**  *(dd mm yy)* | **Stop date**  (tick if □ ongoing)  *(dd mm yy)* | **Severity** |
| --- | --- | --- | --- |
|  | __ /__ /__ | __ /__ /__ □ |  |
|  | __ /__ /__ | __ /__ /__ □ |  |
|  | __ /__ /__ | __ /__ /__ □ |  |
|  | __ /__ /__ | __ /__ /__ □ |  |
|  | __ /__ /__ | __ /__ /__ □ |  |

**Note:** 1=Mild, 2=Moderate, 3=Severe, 4=Life-threatening

1. **Concomitant medication**: new medication or dose change

1□ Yes (If yes, please specify below) 0□ No

| **Medication Name** | **Start date**  *(dd mm yy)* | **Stop date**  (tick if □ ongoing)  *(dd mm yy)* |
| --- | --- | --- |
|  | __ /__ /__ | __ /__ /__ □ |
|  | __ /__ /__ | __ /__ /__ □ |
|  | __ /__ /__ | __ /__ /__ □ |
|  | __ /__ /__ | __ /__ /__ □ |
|  | __ /__ /__ | __ /__ /__ □ |

**6. Physical examination** (Tick box if normal, describe if abnormal)

□ General appearance ______________ □ Skin _________________________

□ Eyes ___________________________ □ Ear, nose, throat _______________

□ Cardiovascular ___________________ □ Respiratory __________________

□ Gastrointestinal __________________ □ Neurological _________________

□ Musculoskeletal __________________ □ Others: _____________________

**7. Pills count**: |_|_| tabs

1. **Laboratory monitoring**

1) Aspartate aminotransferase (AST) |_|_|_|_| U/L

Alanine aminotransaminase (ALT) |_|_|_|_| U/L

2) Lipid profiles

Total cholesterol (TC) |_|_|_|_| mg/dL

Triglyceride (TG) |_|_|_|_| mg/dL

Low Density Lipoprotein (LDL) |_|_|_|_| mg/dL

High Density Lipoprotein (HDL) |_|_|_|_| mg/dL

3) Creatinine kinase (If complaint) |_|_|_|_| U/L

1. **Note**

**________________________________________________________________________________________________________________________________**

Follow up date: |_|_| |_|_| |_|_|

*dd mm yy*

Week 18

PID: |_|_|

Date of visit: |_|_|-|_|_|-|_|_|

*dd*  *mm* *yy*

1. **Medical history**

**___________________________________________________________________________________________________________________________________________________________________________________________________________________________________________________________________________________________________________________________________**

1. **Vital signs**

Body weight (BW): |_|_|_|.|_| kg

Height: |_|_|_|.|_| cm

Body Mass Index (BMI): |_|_|.|_| kg/m^2^

1. **HIV-related illnesses**

| **Diagnosis** | **Start date**  *(dd mm yy)* | **Stop date**  (tick if □ ongoing)  *(dd mm yy)* |
| --- | --- | --- |
|  | __ /__ /__ | __ /__ /__ □ |
|  | __ /__ /__ | __ /__ /__ □ |
|  | __ /__ /__ | __ /__ /__ □ |
|  | __ /__ /__ | __ /__ /__ □ |
|  | __ /__ /__ | __ /__ /__ □ |

1. **Adverse events (AEs)**

1□ Yes (If yes, please specify below) 0□ No

| **Description** | **Start date**  *(dd mm yy)* | **Stop date**  (tick if □ ongoing)  *(dd mm yy)* | **Severity** |
| --- | --- | --- | --- |
|  | __ /__ /__ | __ /__ /__ □ |  |
|  | __ /__ /__ | __ /__ /__ □ |  |
|  | __ /__ /__ | __ /__ /__ □ |  |
|  | __ /__ /__ | __ /__ /__ □ |  |
|  | __ /__ /__ | __ /__ /__ □ |  |

**Note:** 1=Mild, 2=Moderate, 3=Severe, 4=Life-threatening

1. **Concomitant medication**: new medication or dose change

1□ Yes (If yes, please specify below) 0□ No

| **Medication Name** | **Start date**  *(dd mm yy)* | **Stop date**  (tick if □ ongoing)  *(dd mm yy)* |
| --- | --- | --- |
|  | __ /__ /__ | __ /__ /__ □ |
|  | __ /__ /__ | __ /__ /__ □ |
|  | __ /__ /__ | __ /__ /__ □ |
|  | __ /__ /__ | __ /__ /__ □ |
|  | __ /__ /__ | __ /__ /__ □ |

**6. Physical examination** (Tick box if normal, describe if abnormal)

□ General appearance ______________ □ Skin _________________________

□ Eyes ___________________________ □ Ear, nose, throat _______________

□ Cardiovascular ___________________ □ Respiratory __________________

□ Gastrointestinal __________________ □ Neurological _________________

□ Musculoskeletal __________________ □ Others: _____________________

**7. Pills count**: |_|_| tabs

1. **Laboratory monitoring**

1) Aspartate aminotransferase (AST) |_|_|_|_| U/L

Alanine aminotransaminase (ALT) |_|_|_|_| U/L

2) Lipid profiles

Total cholesterol (TC) |_|_|_|_| mg/dL

Triglyceride (TG) |_|_|_|_| mg/dL

Low Density Lipoprotein (LDL) |_|_|_|_| mg/dL

High Density Lipoprotein (HDL) |_|_|_|_| mg/dL

3) Creatinine kinase (If complaint) |_|_|_|_| U/L

1. **Note**

**________________________________________________________________________________________________________________________________**

Follow up date: |_|_| |_|_| |_|_|

*dd mm yy*

Week 22

PID: |_|_|

Date of visit: |_|_|-|_|_|-|_|_|

*dd*  *mm* *yy*

1. **Medical history**

**___________________________________________________________________________________________________________________________________________________________________________________________________________________________________________________________________________________________________________________________________**

1. **Vital signs**

Body weight (BW): |_|_|_|.|_| kg

Height: |_|_|_|.|_| cm

Body Mass Index (BMI): |_|_|.|_| kg/m^2^

1. **HIV-related illnesses**

| **Diagnosis** | **Start date**  *(dd mm yy)* | **Stop date**  (tick if □ ongoing)  *(dd mm yy)* |
| --- | --- | --- |
|  | __ /__ /__ | __ /__ /__ □ |
|  | __ /__ /__ | __ /__ /__ □ |
|  | __ /__ /__ | __ /__ /__ □ |
|  | __ /__ /__ | __ /__ /__ □ |
|  | __ /__ /__ | __ /__ /__ □ |

1. **Adverse events (AEs)**

1□ Yes (If yes, please specify below) 0□ No

| **Description** | **Start date**  *(dd mm yy)* | **Stop date**  (tick if □ ongoing)  *(dd mm yy)* | **Severity** |
| --- | --- | --- | --- |
|  | __ /__ /__ | __ /__ /__ □ |  |
|  | __ /__ /__ | __ /__ /__ □ |  |
|  | __ /__ /__ | __ /__ /__ □ |  |
|  | __ /__ /__ | __ /__ /__ □ |  |
|  | __ /__ /__ | __ /__ /__ □ |  |

**Note:** 1=Mild, 2=Moderate, 3=Severe, 4=Life-threatening

1. **Concomitant medication**: new medication or dose change

1□ Yes (If yes, please specify below) 0□ No

| **Medication Name** | **Start date**  *(dd mm yy)* | **Stop date**  (tick if □ ongoing)  *(dd mm yy)* |
| --- | --- | --- |
|  | __ /__ /__ | __ /__ /__ □ |
|  | __ /__ /__ | __ /__ /__ □ |
|  | __ /__ /__ | __ /__ /__ □ |
|  | __ /__ /__ | __ /__ /__ □ |
|  | __ /__ /__ | __ /__ /__ □ |

**6. Physical examination** (Tick box if normal, describe if abnormal)

□ General appearance ______________ □ Skin _________________________

□ Eyes ___________________________ □ Ear, nose, throat _______________

□ Cardiovascular ___________________ □ Respiratory __________________

□ Gastrointestinal __________________ □ Neurological _________________

□ Musculoskeletal __________________ □ Others: _____________________

**7. Pills count**: |_|_| tabs

1. **Laboratory monitoring**

1) Aspartate aminotransferase (AST) |_|_|_|_| U/L

Alanine aminotransaminase (ALT) |_|_|_|_| U/L

2) Lipid profiles

Total cholesterol (TC) |_|_|_|_| mg/dL

Triglyceride (TG) |_|_|_|_| mg/dL

Low Density Lipoprotein (LDL) |_|_|_|_| mg/dL

High Density Lipoprotein (HDL) |_|_|_|_| mg/dL

3) Creatinine kinase (If complaint) |_|_|_|_| U/L

1. **Note**

**________________________________________________________________________________________________________________________________**

Follow up date: |_|_| |_|_| |_|_|

*dd mm yy*

Week 26

PID: |_|_|

Date of visit: |_|_|-|_|_|-|_|_|

*dd*  *mm* *yy*

1. **Medical history**

**___________________________________________________________________________________________________________________________________________________________________________________________________________________________________________________________________________________________________________________________________**

1. **Vital signs**

Body weight (BW): |_|_|_|.|_| kg

Height: |_|_|_|.|_| cm

Body Mass Index (BMI): |_|_|.|_| kg/m^2^

1. **HIV-related illnesses**

| **Diagnosis** | **Start date**  *(dd mm yy)* | **Stop date**  (tick if □ ongoing)  *(dd mm yy)* |
| --- | --- | --- |
|  | __ /__ /__ | __ /__ /__ □ |
|  | __ /__ /__ | __ /__ /__ □ |
|  | __ /__ /__ | __ /__ /__ □ |
|  | __ /__ /__ | __ /__ /__ □ |
|  | __ /__ /__ | __ /__ /__ □ |

1. **Adverse events (AEs)**

1□ Yes (If yes, please specify below) 0□ No

| **Description** | **Start date**  *(dd mm yy)* | **Stop date**  (tick if □ ongoing)  *(dd mm yy)* | **Severity** |
| --- | --- | --- | --- |
|  | __ /__ /__ | __ /__ /__ □ |  |
|  | __ /__ /__ | __ /__ /__ □ |  |
|  | __ /__ /__ | __ /__ /__ □ |  |
|  | __ /__ /__ | __ /__ /__ □ |  |
|  | __ /__ /__ | __ /__ /__ □ |  |

**Note:** 1=Mild, 2=Moderate, 3=Severe, 4=Life-threatening

1. **Concomitant medication**: new medication or dose change

1□ Yes (If yes, please specify below) 0□ No

| **Medication Name** | **Start date**  *(dd mm yy)* | **Stop date**  (tick if □ ongoing)  *(dd mm yy)* |
| --- | --- | --- |
|  | __ /__ /__ | __ /__ /__ □ |
|  | __ /__ /__ | __ /__ /__ □ |
|  | __ /__ /__ | __ /__ /__ □ |
|  | __ /__ /__ | __ /__ /__ □ |
|  | __ /__ /__ | __ /__ /__ □ |

**6. Physical examination** (Tick box if normal, describe if abnormal)

□ General appearance ______________ □ Skin _________________________

□ Eyes ___________________________ □ Ear, nose, throat _______________

□ Cardiovascular ___________________ □ Respiratory __________________

□ Gastrointestinal __________________ □ Neurological _________________

□ Musculoskeletal __________________ □ Others: _____________________

1. **Pills count**: |_|_| tabs
2. **Laboratory monitoring**

1) Aspartate aminotransferase (AST) |_|_|_|_| U/L

Alanine aminotransaminase (ALT) |_|_|_|_| U/L

2) Lipid profiles

Total cholesterol (TC) |_|_|_|_| mg/dL

Triglyceride (TG) |_|_|_|_| mg/dL

Low Density Lipoprotein (LDL) |_|_|_|_| mg/dL

High Density Lipoprotein (HDL) |_|_|_|_| mg/dL

3) Creatinine kinase |_|_|_|_| U/L

4) ATV level |_|_|_|.|_| mcg/L

1. **Note**

**________________________________________________________________________________________________________________________________**

Follow up date: |_|_| |_|_| |_|_|

*dd mm yy*

**Measurement**

| **Weeks** | **Screening visit** | **4** | **8** | **12** | **14** | **18** | **22** | **26** |
| --- | --- | --- | --- | --- | --- | --- | --- | --- |
| AEs |  |  |  |  |  |  |  |  |
| Lipid profiles   - TC - TG - LDL - HDL |  |  |  |  |  |  |  |  |
| AST  ALT |  |  |  |  |  |  |  |  |
| FBS |  |  |  |  |  |  |  |  |
| Cr |  |  |  |  |  |  |  |  |
| ATV level |  |  |  |  |  |  |  |  |
| CPK |  |  |  |  |  |  |  |  |
| Statin level |  |  |  |  |  |  |  |  |
| Immunologic study/  miscellaneous |  |  |  |  |  |  |  |  |
